# Association between amorphous calcium-phosphate ratios in circulating calciprotein particles and prognostic biomarkers in hemodialysis patients

**DOI:** 10.1038/s41598-022-17405-7

**Published:** 2022-07-29

**Authors:** Kimihiko Nakamura, Naohito Isoyama, Yuki Nakayama, Toshiya Hiroyoshi, Koki Fujikawa, Yutaka Miura, Hiroshi Kurosu, Hideyasu Matsuyama, Makoto Kuro-o

**Affiliations:** 1 Department of Urology, Graduate School of Medicine, Yamaguchi University, 1‑1‑1, Minamikogushi, Ube, Yamaguchi, 755-8505 Japan; 2 Division of Anti-Aging Medicine, Center for Molecular Medicine, Jichi Medical University, 3311-1 Yakushiji, Shimotsuke, Tochigi 329-0498 Japan

**Keywords:** Renal replacement therapy, Prognostic markers

## Abstract

Calciprotein particles (CPPs) are circulating colloidal mineral-protein complexes containing crystalline and/or non-crystalline (amorphous) calcium-phosphate (CaPi). Serum CPP levels correlate with vascular stiffness and calcification in patients with chronic kidney disease (CKD). In vitro studies showed that CPPs containing crystalline CaPi were more arteriosclerogenic and inflammogenic than CPPs without containing crystalline CaPi. Thus, we hypothesized that not only the quantity but also the quality of CPPs (the phase of CaPi) might affect clinical outcomes. To test this hypothesis, we quantified amorphous CaPi ratio defined as the ratio of the amorphous CaPi amount to the total CaPi amount in serum CPPs from 183 hemodialysis patients and explored its possible correlation with serum parameters associated with prognosis of hemodialysis patients. Multivariate analysis revealed that the amorphous CaPi ratio correlated positively with hemoglobin and negatively with fibroblast growth factor-21 (FGF21), which remained significant after adjusting for the total CaPi amount. Because low hemoglobin and high FGF21 are associated with increased mortality, the present study warrants further studies to determine whether low amorphous CaPi ratio in circulating CPPs may be associated with poor prognosis in hemodialysis patients.

## Introduction

Calciprotein particles (CPPs) are colloidal mineral-protein complexes mainly composed of solid-phase calcium phosphate (CaPi) and serum protein fetuin-A^1,2^. Formation of CPPs is a physicochemical phenomenon that progresses spontaneously over time in solution containing calcium, phosphate, and serum. When the concentration of calcium and phosphate ions exceeds the solubility limit, precipitates of amorphous CaPi are generated, which are adsorbed by fetuin-A and prevented from further growth. Consequently, fetuin-A molecules laden with tiny amorphous CaPi are generated. These particles are termed primary CPPs. Primary CPPs spontaneously undergo self-aggregation and phase transition of CaPi from the amorphous phase to the crystalline phase to become secondary CPPs^3^. Secondary CPPs have the activity that induces calcification in cultured vascular smooth muscle cells and innate immune responses in cultured macrophages, whereas primary CPPs do not have such pathogenic activity^4,5^.

CPPs circulate in the blood as colloids. Recent clinical studies demonstrated that serum CPP levels were increased with decline of renal function and correlated with clinical parameters for inflammation and vascular calcification/stiffness in patients with chronic kidney disease (CKD)^6,7^. Considering the activity of secondary CPPs in cultured cells, the correlation observed in these clinical studies may be causation. Namely, secondary CPPs containing crystalline CaPi may be a pathogen of vascular calcification and chronic non-infectious inflammation in CKD patients. Because any pathogenic activity has not been observed in primary CPPs in vitro, we reason that not only the amount of CPPs but also the phase of CaPi in the CPPs may determine pathogenic activity of circulating CPPs and thus clinical outcomes of CKD patients. Specifically, we hypothesize that higher amorphous CaPi ratio, defined as the ratio of the amorphous CaPi amount to the total CaPi amount in CPPs, may be associated with better prognosis. To test this hypothesis, we have developed an assay to estimate the amorphous CaPi ratio in serum CPPs and determined whether it may be associated with serum parameters reported to correlate with prognosis in hemodialysis patients.

## Results

**Figure 1:**
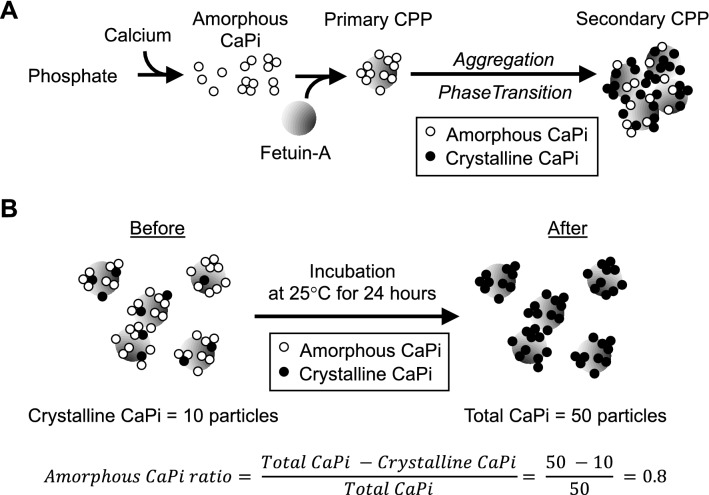
Calciprotein particles (CPPs) with different properties. (**A**) A schematic representation of CPP formation and maturation. Precipitates of amorphous CaPi (open circles) are adsorbed by fetuin-A protein (grey circles) to form primary CPPs. Primary CPPs undergo aggregation and transition of CaPi from the amorphous phase to the crystalline phase to become secondary CPPs. (**B**) A schematic representation of estimation of the amorphous CaPi ratio. Incubation at 25 °C for 24 h induces amorphous-to-crystalline phase transition of CaPi. Because the gel filtration assay can measure crystalline CaPi but not amorphous CaPi, the amorphous CaPi amount (40; the number of open circles) can be estimated as the difference in the number of closed circles between before (10) and after (50) the incubation.

The CPP assay used in this study, termed “the gel filtration assay”, measures the amount of crystalline CaPi in serum CPPs, because the assay uses a fluorescent bisphosphonate (OsteoSense) as a probe, which binds to crystalline CaPi but not to amorphous CaPi^8,9^. The amount of amorphous CaPi in serum CPPs was estimated as follows. Immediately after thawing a serum sample, a part of the sample was used for measuring the crystalline CaPi amount by the gel filtration assay. The rest of the serum sample was incubated at 25 °C for 24 h to convert the amorphous CaPi in the CPPs to crystalline CaPi in vitro and then subjected to the gel filtration assay to determine the total CaPi amount (the sum of crystalline CaPi and amorphous CaPi). The difference between before and after the incubation at 25 °C for 24 h was defined as the amorphous CaPi amount. The amorphous CaPi ratio was defined as the ratio of the amorphous CaPi amount to the total CaPi amount (Fig. 1).

Clinical and biochemical characteristics of the 183 hemodialysis patients are summarized in Table 1. All the four parameters of CPPs (total CaPi, crystalline CaPi, amorphous CaPi, and amorphous CaPi ratio) were log-transformed before the statistical analysis because of their skewed distribution. In univariate analysis, total CaPi was correlated positively with hemoglobin, creatinine, albumin, calcium, and phosphate, and negatively with age, BAP, FGF21, and CRP (Table 2). Crystalline CaPi was correlated positively with albumin, calcium, and phosphate, and negatively with age and BAP (Table 3). Amorphous CaPi was correlated positively with hemoglobin, creatinine, albumin, calcium, and phosphate, and negatively with age, BAP, FGF21, and CRP (Table 4). Amorphous CaPi ratio was correlated positively with total CaPi, hemoglobin, creatinine, albumin, calcium, and phosphate, and negatively with BAP, FGF21, and CRP (Table 5).

**Table 1:** Clinical and laboratory characteristics of the participants.

	n = 183
Age (year)	68 ± 13
Sex male (%)	105 (57.6)
BMI (kg/m^2^)	20.7 ± 3.5
SBP (mmHg)	144 ± 25
DBP (mmHg)	79 ± 13
Period of hemodialysis (months)	71 (31–142)
Kt/v	1.83 ± 0.35
Serum albumin (g/dl)	3.5 (3.3–3.8)
Serum Creatinine (mg/dl)	7.7 (6.3–9.0)
Serum Ca (mg/dl)	8.4 (7.8–8.8)
Serum P (mg/dl)	5.0 (4.1–5.7)
iPTH (pg/ml)	151.0 (81.0–207.8)
BAP (µg/l)	14.9 (11.5–20.7)
Ferritin (ng/ml)	91.0 (49.9–181.9)
TSAT (%)	27 (19–35)
CRP (mg/dl)	0.2 (0.1–0.4)
**CPP**	
Crystalline CaPi (AU)	7922 (5381–12,204)
Total CaPi (AU)	39,584 (19,075–67,576)
Amorphous CaPi (AU)	32,047 (12,619–54,245)
Amorphous CaPi ratio	0.77 (0.65–0.83)

Data are expressed as mean ± SD or number or median (interquartile range). *BMI* body mass index, *SBP* systolic blood pressure, *DBP* diastolic blood pressure, *iPTH* intact parathyroid hormone, *BAP* bone specific alkaline phosphatase, *TSAT* transferrin saturation, *CRP* C-reactive protein.

**Table 2:** Univariate and multivariate analysis between total CaPi amounts and serum parameters in the hemodialysis patients. Significant values are in bold.

Variable	Univariate		Multivariate	
	R	P-value	β	P-value
Age	− 0.1816	**0.0150**	− 0.0195	0.7902
BMI	0.0716	0.3562	–	–
Duration of dialysis	− 0.0106	0.8870	–	–
Hemoglobin	0.1613	**0.0300**	0.0288	0.6868
Creatinine	0.2212	**0.0028**	0.0712	0.3170
Albumin	0.2510	**0.0007**	0.0430	0.5995
Calcium	0.3220	**< 0.0001**	0.2129	**0.0031**
Phosphate	0.4425	**< 0.0001**	0.3375	**< 0.0001**
Intact PTH	0.0275	0.7141	–	–
BAP	− 0.2401	**0.0013**	− 0.0508	0.4771
TRACP-5b	− 0.0707	0.3442	–	–
TSAT	− 0.5070	0.4514	–	–
FGF21	− 0.2548	**0.0005**	− 0.1671	**0.0342**
CRP	− 0.1616	**0.0302**	0.0592	0.4402

*R* coefficient of determination, *β* standardized partial regression coefficient.

**Table 3:** Univariate and multivariate analysis between crystalline CaPi amounts and serum parameters in the hemodialysis patients. Significant values are in bold.

Variable	Univariate		Multivariate	
	R	P-value	β	P-value
Age	− 0.2163	**0.0034**	− 0.8821	0.2422
BMI	− 0.0202	0.7949	–	–
Duration of dialysis	0.0835	0.2639	–	–
Hemoglobin	− 0.0383	0.6035	–	–
Creatinine	0.0996	0.1835	–	–
Albumin	0.1626	**0.0288**	0.0051	0.4999
Calcium	0.2027	**0.0062**	0.1470	0.1110
Phosphate	0.3532	**< 0.0001**	0.2863	**0.0002**
Intact PTH	0.0112	0.8818	–	–
BAP	− 0.1750	**0.0198**	− 0.0436	0.5616
TRACP-5b	− 0.1149	0.1236	–	–
TSAT	− 0.1082	0.1517	–	–
FGF21	− 0.1249	0.5098	–	–
CRP	− 0.1384	0.0639	–	–

*BMI* body mass index, *PTH* parathyroid hormone, *BAP* bone specific alkaline phosphatase, *TRACP-5b* tartrate-resistant acid phosphatase-5b, *TSAT* transferrin saturation, *CRP* C-reactive protein.

**Table 4:** Univariate and multivariate analysis between amorphous CaPi amounts and serum parameters in the hemodialysis patients. Significant values are in bold.

Variable	Univariate		Multivariate	
	R	P-value	β	P-value
Age	− 0.1493	**0.0048**	0.0046	0.9502
BMI	0.0890	0.2513	–	–
Duration of dialysis	− 0.0356	0.6346	–	–
Hemoglobin	0.1978	**0.0076**	0.0754	0.2569
Creatinine	0.2288	**0.0020**	0.0819	0.2536
Albumin	0.2549	**0.0008**	0.0345	0.6759
Calcium	0.3171	**< 0.0001**	0.2188	**0.0026**
Phosphate	0.4151	**< 0.0001**	0.3085	**< 0.0001**
Intact PTH	0.0288	0.7013	–	–
BAP	− 0.2300	**0.0021**	− 0.0451	0.5306
TRACP-5b	− 0.0501	0.5033	–	–
TSAT	− 0.0359	0.6353	–	–
FGF21	− 0.2608	**0.0004**	− 0.1775	**0.0257**
CRP	− 0.1489	**0.0460**	0.0769	0.3206

**Table 5:** Univariate and multivariate analysis between amorphous CaPi ratios and serum parameters in the hemodialysis patients. Significant values are in bold.

Variable	Univariate		Multivariate	
	R	P-value	β	P-value
Age	− 0.0748	0.3172	–	–
BMI	− 0.0738	0.3351	–	–
Duration of dialysis	− 0.0519	0.4877	–	–
Hemoglobin	0.2669	**0.0001**	0.1584	**0.0246**
Creatinine	0.1965	**0.0082**	0.0692	0.3094
Albumin	0.2840	**0.0001**	0.0928	0.2394
Calcium	0.2068	**0.0048**	0.0373	0.5961
Phosphate	0.2368	**0.0013**	0.0428	0.5043
Intact PTH	0.2010	0.7793	–	–
BAP	− 0.2791	**0.0002**	0.1957	0.1022
TRACP-5b	0.0518	0.4888	–	–
TSAT	0.0122	0.8720	–	–
FGF21	− 0.3158	**0.0004**	− 0.1886	**0.0155**
CRP	− 0.1596	**0.0234**	0.0614	0.4160
Total CaPi	0.5048	**< 0.0001**	0.0049	**< 0.0001**

**Figure 2:**
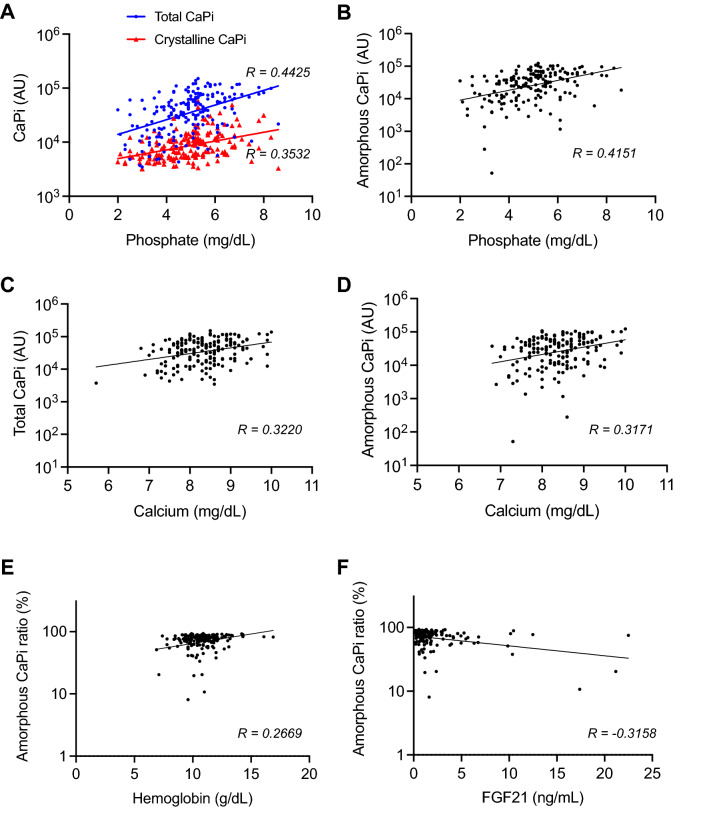
Scattered plots between CPP and other serum parameters. Correlation coefficient (R) is indicated. Association of serum phosphate levels with total (**A**, blue), crystalline (**A**, red), and amorphous CaPi (**B**) amounts. Association of serum calcium levels with total (**C**) and amorphous (**D**) CaPi amounts. Association of amorphous CaPi ratios with hemoglobin (**E**) and FGF21 (**F**).

Among these parameters, serum phosphate and calcium levels remained significantly correlated with total CaPi (Table 2), crystalline CaPi (Table 3), and amorphous CaPi (Table 4) in multivariate analysis adjusted by all the parameters identified as significantly correlated in univariate analysis. The amorphous CaPi ratio was correlated positively with hemoglobin and negatively with FGF21 after adjusting for the total CaPi (Table 5). The scattered plots of the parameters that remained significantly correlated in multivariate analysis were shown in Fig. 2.

## Discussion

Serum FGF21 levels are consistently increased during CKD progression since early stages^10,11^. FGF21 is a member of the fibroblast growth factor (FGF) subfamily that functions as an endocrine factor^12^. A characteristic feature of FGF21 lies in the fact that it requires βKlotho, a single-pass transmembrane protein, to bind to FGF receptor-1c (FGFR1c)^13,14^. Namely, the physiological receptor for FGF21 is not an FGF receptor but a binary complex of FGFR1c and βKlotho, which is expressed predominantly in adipocytes and neurons in the suprachiasmatic nucleus (SCN)^15^. In response to various types of stress including fasting, FGF21 is secreted from hepatocytes and acts directly on adipocytes and indirectly on the liver to induce metabolic responses to fasting^16,17^. In addition, FGF21 passes through the blood–brain barrier and acts directly on SCN neurons to activate the sympathetic nervous system and the hypothalamus–pituitary–adrenal axis, thereby inducing responses to stress^18^. The increase in FGF21 during CKD progression can be viewed as a survival response, because CKD mice lacking FGF21 have shorter lifespan than wild-type CKD mice^19^. Thus, high serum FGF21 levels may indicate a state of intense stress and is indeed associated with poor prognosis in hemodialysis patients^20^. Conversely, the fact that the hemodialysis patients with higher amorphous CaPi ratios had lower serum FGF21 levels (Table 5) suggests that they may have better prognosis. This notion is also supported by the fact that the amorphous CaPi ratio was positively correlated with hemoglobin, because higher hemoglobin was reported to be associated with better prognosis in CKD patients^21^.

Serum FGF23 levels were reported to correlate with mortality independently of serum phosphate levels in hemodialysis patients^22^. Although we were unable to measure FGF23 in this study due to the limitation of the sample volume, we speculate that FGF23 may correlate positively with amorphous and/or crystalline CaPi contents, because CPPs stimulated FGF23 secretion/production in cultured osteoblastic cells^23^. Indeed, we previously reported that high crystalline CaPi amounts were associated with high serum FGF23 levels in hemodialysis patients^24^. Chronic non-infectious inflammation is also known to be associated with poor prognosis in hemodialysis patients^25^. However, we were unable to detect significant correlation between CRP and any types of CPPs in multivariate analysis in this study.

Serum phosphate levels were positively associated not only with amorphous CaPi amounts but also crystalline CaPi amounts (Fig. 2A,B). Crystalline CaPi amounts reflect secondary CPPs with pathogenic activity that induces calcification in cultured vascular smooth muscle cells, whereas amorphous CaPi amounts reflect primary CPPs. There is no experimental evidence showing that primary CPPs exert the pathogenic activity like secondary CPPs or counteract the pathogenic activity of secondary CPPs. Therefore, we assume that secondary CPPs, but not primary CPPs, may contribute to poor clinical outcomes associated with hyperphosphatemia.

In general, insoluble materials such as lipids and CaPi are bound to specific serum proteins to form colloidal particles and dispersed in the blood to be transported between organs through systemic circulation. Lipids are bound to apoproteins to form colloidal particles called lipoproteins. The activity of lipoproteins depends on their composition and physical properties, as evidenced by the fact that low-density lipoprotein (LDL) is pro-atherogenic, whereas high-density lipoprotein (HDL) is anti-atherogenic^26^. Thus, not only total cholesterol levels but also lipoprotein fractions have been measured for evaluating the risk for atherosclerosis. Similarly, insoluble CaPi is bound to fetuin-A to form colloidal particles called CPPs. The activity of CPPs depends on their composition and physical properties, as evidenced by the fact that secondary CPPs, but not primary CPPs, exert cytotoxic activity^27^. Thus, we propose that measurement of both quantity (serum levels) and quality (amorphous CaPi ratios) of CPPs may be informative for evaluating prognosis and clinical outcomes in hemodialysis patients.

One of the limitations in this study is lack of a cohort of healthy individuals. Unlike in hemodialysis patients, CPP levels were barely increased after incubation at 25 °C for 24 h in healthy volunteers^8^. Therefore, we presume that measurement of amorphous CaPi amounts and amorphous CaPi ratios using the gel filtration assay may not be feasible in healthy individuals. Other limitations include a cross-sectional design and a small number of participants. Further long-term, large-scale, prospective cohort studies are needed to conclude that the amorphous CaPi ratio serves as an independent parameter that predicts prognosis in hemodialysis patients.

## Methods

### Patients

Total of 183 end-stage renal disease (ESRD) patients receiving hemodialysis (57.6% men, median age 71, range 41–100 years) were recruited in a single hospital (St. Hill hospital, Ube, Yamaguchi, Japan). The exclusion criteria were active malignancy and severe infectious disease. Patient’s case history and comorbidities were obtained from medical records. The causes of kidney disease included diabetic nephropathy (33%), hypertensive nephrosclerosis (24%), chronic glomerular nephritis (20%), polycystic kidney disease (2%), and others (21%). Comorbidities included hypertension (82%), diabetes mellitus (39%), cardiovascular disease defined as history of cardiac and cerebrovascular (including stroke) events (40%). The study was conducted in accordance with the Declaration of Helsinki. The research protocol was approved by the Medical Ethics Committees of St. Hill hospital. All the patients provided their written informed consent.

### Data collection

Blood samples were collected from patients immediately before starting a hemodialysis session. Serum FGF21 levels were measured using a sandwich enzyme-linked immunosorbent assay (ELISA) kit (Bio Vender, Mpdrice, Czech Republic) in accordance with the manufacturer’s instructions. Other laboratory data were measured using certified methods at the Department of Clinical Chemistry of the hospital.

### Measurement of serum CPP levels

Serum CPP levels were measured by the gel filtration assay as we reported previously^8^. Briefly, serum and OsteoSense 680EX (PerkinElmer), a near-infrared fluorescent probe that binds to crystalline CaPi, was added to Dulbecco’s Modified Eagle Medium (DMEM) containing 100 mM HEPES (pH 8.0). After incubation at 25 °C for 60 min, the mixture was applied to a gel filtration spin column (Bio-rad, molecular weight cut-off 40 kDa) and centrifuged at 1000*g* for 2 min. The flow-through was diluted with the same volume of 2% SDS and 100 mM EDTA and subjected to quantification of the OsteoSense fluorescence using a scanner (Odyssey CLx, LI-COR, excitation at 685 nm, emission at 700 nm).

### Statistical analysis

All statistical analyses were performed by using JMP Pro 14 software program (SAS Institute, Cary, NC, USA). Significant values were determined via Student’s *t* test and P < 0.05 was considered significant. Spearman rank correlation was performed to determine correlations with continuous variables.
